# Plasma lipidomic patterns associated with disease activity in chronic inflammatory demyelinating polyradiculoneuropathy (LIPID-CIDP)

**DOI:** 10.1016/j.jlr.2025.100903

**Published:** 2025-09-17

**Authors:** Kristina auf dem Brinke, Lisa-Marie Borsch, Christian Klose, Jana Zschüntzsch, Liza Vinhoven, Manuel Nietert, Seyed Siyawasch Justus Lattau, Dirk Fitzner

**Affiliations:** 1 Department of Neurology, University Medical Center Göttingen, Göttingen, Germany; 2 Lipotype GmbH, Dresden, Germany; 3 Neuromuscular Center Göttingen, Department of Neurology, University Medical Center Göttingen, Göttingen, Germany; 4 Department of Medical Bioinformatics, University Medical Center Göttingen, Göttingen, Germany

**Keywords:** Lipidomics, Lipid droplets, Inflammation, Lipotoxicity, Phospholipids/ Metabolism, Polyradiculoneuropathy, Chronic Inflammatory Demyelinating, Lipid Metabolism, Membrane Lipids

## Abstract

Chronic inflammatory demyelinating polyneuropathy (CIDP) is an immune-mediated neuropathy that causes significant disability in patients. Although pathogenic mechanisms remain unclear, it is known that inflammation results in segmental demyelination. This study aims to investigate the plasma lipidomic profile of patients with CIDP to identify lipid patterns associated with disease activity. Using high-throughput shotgun lipidomics, we analyzed and compared the plasma lipidome of 30 patients with CIDP (mean age ± SD: 60.7 ± 12.2 years) with that of 30 individuals diagnosed with non-demyelinating neurological disorders (OND; mean age ± SD: 52.8 ± 10.3 years). Lipids were quantified in absolute [pmol] and relative concentrations [mol%], and their levels were correlated with CIDP disease activity and clinical disability scores (R-ODS, INCAT and MRC). To control for confounders such as age and weight, strongly correlated lipids were excluded. The analysis identified 669 molecular lipid species across 15 lipid classes, revealing a significant elevation in the diacylglycerol (DAG) class in CIDP patients. Furthermore, specific lipid subspecies, including triacylglycerol (TAG), DAG, and ether-linked phosphatidylcholine (PC O-), were significantly correlated with disease activity. A set of distinct lipid subspecies, including phosphatidylcholine (PC), lyso-phosphatidylcholine (LPC), phosphatidylinositol (PI), sphingomyelin (SM), and cholesterol ester (CE), showed strong associations with clinical disability scores. These findings suggest that CIDP is characterized by distinct lipidomic profiles modulated by disease activity. This dataset could pave the way for future studies in larger cohorts evaluating the potential of plasma lipid profiles to serve as biomarkers for disease activity and severity, aiding in informing clinical management.

Chronic inflammatory demyelinating polyradiculoneuropathy (CIDP) is the most common chronic immune-mediated neuropathy with a prevalence of around 2.81 per 100.000 (1). It is characterized by progressive muscle weakness and sensory dysfunction, affecting gait and activities of daily living (2). The clinical presentation of CIDP is diverse, and a combination of clinical and electrodiagnostic characteristics as well as response to treatment are currently the most widely accepted criteria to confirm the diagnosis of CIDP (3, 4). Treatment options for CIDP include immunoglobulins, corticosteroids, plasmapheresis, and neonatal Fc-receptor inhibitors (5, 6). Remission of the disease is possible; however, most patients with CIDP require maintenance therapy for years or even decades (5). CIDP is therefore having a considerable impact on patients’ autonomy and patient-related health care costs (7).

Although a few studies have attempted on finding biomarkers aiding to diagnose and assess the clinical activity of CIDP, so far clinicians are essentially relying on clinical parameters regarding diagnostic and therapeutic decisions. By combining clinical symptoms and evidence of demyelination on nerve conduction tests as well as excluding other causes, the widely used EFNS/PNS(3) or newly revised EAN/PNS(4) criteria exhibit a high sensitivity and specificity; nevertheless, delayed diagnoses and misdiagnoses are common (8, 9). In addition, the existing criteria are not suitable to assess disease activity. Consequently, clinical decisions during routine practice regarding immunotherapies are not based on objective metrics but instead rely on a clinician’s interpretation of a patient’s subjective experience (8).

The abiding theory of CIDP pathogenesis is that humoral and cell-mediated mechanisms are directed against yet undefined Schwann-cell or myelin antigens and lead to segmental demyelination (10, 11). In detail, myelin is a lipid-rich and multilamellar structure that encloses segments of axons in the central (CNS) and peripheral nervous systems (PNS). It is formed by oligodendrocytes (CNS) and Schwann-cells (PNS). While the protein composition differs substantially in PNS and CNS myelin, lipid species are remarkably similar (12). Lipids account for at least 70% of the dry weight of myelin membranes (13). In a demyelinating process, these lipids are released into the extracellular space. As a consequence, the lipid-rich myelin debris is taken up and processed by inflammatory cells, including neutrophils and macrophages, while at the same time having proinflammatory effects (14).

To provide a better understanding of the inflammatory and demyelinating process, lipidomic approaches have been discussed as a powerful tool to identify lipid molecules associated with pathological conditions recently. Previous studies have reported distinct lipid profiles in the plasma of patients with demyelinating diseases such as acute inflammatory demyelinating polyneuropathy (AIDP) and Multiple sclerosis (MS) (15, 16, 17). However, in patients with CIDP, mass-spectroscopy lipidomic analyses of patient samples have not been reported so far, although demyelination, and thus release of lipid-enriched myelin debris with a highly specific lipid composition, is a pathophysiological hallmark of the disease. Moreover, lipidomic analyses could depict different aspects of the disease, such as the inflammatory process, the demyelinating process and the axonal damage.

In the current study, we used a high-throughput state-of-the-art quantitative mass-spectrometry-based shotgun lipidomic platform (18, 19) for blood plasma analysis of patients with CIDP and other neurological diseases. Thus, we identified specific lipid species correlating with the diagnosis of CIDP. Adding on, we performed a subgroup analysis exploring the correlation between CIDP disease activity and our lipidomic analysis to identify a specific subset of lipids that were significantly associated with disease activity. At last, we correlated the lipid profile of CIDP patients with commonly used clinical scores assessing the disability of CIDP patients, to identify a lipidomic pattern that correlates with these validated clinical scores.

## Materials and methods

### Ethics approval and informed consent statement

The Ethics Committee of the University Medical Center Göttingen has approved the analysis conducted in this study (09/10/10). All examinations and experiments were carried out in accordance with the guidelines of the Declaration of Helsinki. All participants were at least 18 years of age and gave written informed consent.

### Patient samples/study cohort

Between March and June 2021, we prospectively enrolled 60 neurological patients suffering from CIDP or other neurological diseases at the University Medical Center Göttingen. Patients were recruited during their routine visits to the outpatient clinic of the University Hospital. CIDP patients were required to fulfill the 2010 revised EFNS criteria (3). The clinical scores R-ODS (20), MRC (21, 22) and INCAT (23) were used to quantify disability at the time of sampling. CDAS-Score was used as a grading system to assess disease activity (24, 25). Patients affected by non-demyelinating neurological diseases were enrolled in a control group named OND. All patients were enrolled independent of disease severity and treatment.

### Lipidomics

Blood samples were collected in the morning, prior to any therapeutic procedures. Blood plasma was isolated by centrifugation (2,000g, 10 min at room temperature) of one collected EDTA-tube. After centrifugation, the plasma phase was transferred and stored at −80 °C. The shotgun nano-electrospray high-resolution Orbitrap mass spectrometry was performed by Lipotype GmbH (Dresden, Germany) as described in (19). This analysis produced two datasets: one containing [mol%] abundances of 669 detected lipid species (eAppendix 1) and another with [pmol] concentrations for 16 lipid classes (eAppendix 2). According to MS-only acquired data, lipids are named as: class name < sum of carbon atoms>:<sum of double bonds>;<sum of hydroxyl groups>. For Example, “TAG 54:8;0” denotes a triacylglycerol whose three fatty acyl chains together contain 54 carbons, 8 double bonds, and no hydroxyl groups. In case of sphingolipids, “SM 34:1;2” denotes a sphingomyelin species with a total of 34 carbon atoms, 1 double bond, and 2 hydroxyl groups in the ceramide backbone.

For lipids where the acyl chains were identified, the annotation contains a specification for each chain instead of the total carbon sum. For example, “DAG 16:0;0_18:1;0 “denotes a diacylglycerol containing a hexadecanoic acyl chain (16:0;0) and an octadecenoic acyl chain (18:1;0). The underscore (“_”) indicates that the sn-1 versus sn-2 positions on the glycerol backbone cannot be resolved. In contrast, “PC O-18:2;0/14:0;0” denotes an ether-phosphatidylcholine in which an 18-carbon alkyl chain with two double bonds (O-18:2;0) is ether-linked at the sn-1 position and a myristoyl acyl chain (14:0;0) is esterified at the sn-2 position; the slash (“/”) signifies that the sn-positions can be resolved. To ensure full compliance with the 2020 Shorthand Nomenclature standard from Liebisch *et al.* (26), we provide a list with all lipid names mapped to Shorthand2020 annotation (eAppendix 14) using Goslin 2.0 (27).

### Statistical analysis

All analysis was conducted in R (v 4.4.0) (28). The following description of statistical analysis was performed on eAppendix 1. The data set was filtered so that each lipid included in the final analysis was measured in at least 10% of all participants (Supplemental Fig. 1). After filtering the initial set of 669 lipids, 444 lipids remained. It is common for shotgun lipidomic datasets to have missing values because abundances may fall below the detection limit (29). Therefore, we imputed missing values with abundances ranging between 0 and the calculated limit of detection for each lipid in each cohort (Supplemental Fig. 2 and eAppendix 3). The dataset was log-scaled and centered, resulting in a visually normal distribution of lipid species (Supplemental Fig. 3) that permitted the use of parametric statistical tests. Subsequently, Pearson correlation was employed to assess the correlation between lipid species and BMI, as well as age. All lipid species with an absolute correlation value greater than 0.5 were acknowledged as relevantly correlated (Supplemental Fig. 4 and eAppendix 4). Principal Component Analysis (PCA) was generated by the stats::prcomp function (Supplemental Fig. 5). Volcano-Plots in Figs. 2 and 4 and eFig. 6 were calculated by Welch’s *t* test. Supervised machine learning was conducted using oPLS-DA. The oPLS-DA was generated via the ropls::opls () function (30) (Supplemental Fig. 6). Prior to calculation, the dataset was divided into two distinct subsets: a training set (60% of the data) and a testing set (40% of the data). To ensure that the distribution of the two sets remained consistent with that of the original dataset, a stratified division was employed. The results of the model fit with 5-fold cross-validation are presented in the eAppendix 5. The top 30 lipid species with the greatest impact on the oPLS-DA classification were identified using the ropls::getVipVn function (Supplemental Fig. 6B). For correlation analysis presented in Fig. 5, stats::cor.test(x, y, method = “pearson”) was used (eAppendix 6).

To assess potential bias toward low-abundance lipids near the limit of detection, we examined the unprocessed mol% values of TAG and DAG species (Supplemental Fig. 9). Lipids were ranked by mean abundance without imputation or transformation. Species identified as significant in Fig. 2 were marked in green, and those highlighted in Fig. 3 in red. This analysis indicates that the selection of significant lipids is not predominantly driven by low-abundance features.

## Results

### Study population

We included 30 CIDP-patients during their routine visits to the outpatient clinic of the Neuromuscular Center of the University Hospital Göttingen, Germany, between March and June 2021. We compared them to 30 patients with other, non-demyelinating neurological diseases (OND), whose data was collected during the same period at the same study site, as described by Lattau *et al.*, 2024. The average age of participants was 60.7 years (±12.2 SD) in the CIDP-group and 52.8 years (±10.3 SD) in the OND-group, with a mean Body Mass Index (BMI) of approximately 28.5 (±4.6 SD) and 27.9 (±6.1 SD) (Table 1). There was a significant age difference between the groups, with overlapping margins of standard deviation.

**Table 1:** Demographic and clinical data of patients with CIDP and disease controls

Demographic Parameter	CIDP (n, 30)	OND (n, 30)	*P*-value	Cohen's d
Sex [Male, %]	66.7%	33.3%	-	-
Age [y, mean ± SD]	60.7 ± 12.2	52.8 ± 10.3	0.009^a^	0.7
BMI [mean ± SD]	28.5 ± 4.6	27.9 ± 6.1	0.562^b^	0.12
INCAT [mean ± SD]	2.7 ± 1.7	-	-	-
R-ODS [mean ± SD]	34.4 ± 11.4	-	-	-
MRC [mean ± SD]	53.3 ± 6.5	-	-	-
CDAS [active/stable]	7/23	-	-	-

Data of OND patients have been published in Lattau *et al.* 2024 (38). Statistical analysis in eAppendix 7.

CIDP = chronic inflammatory demyelinating polyradiculoneuropathy; OND = other neurological diseases; BMI = body mass index; INCAT = inflammatory neuropathy cause and treatment disability score; R-ODS = Rasch-built overall disability scale; MRC = medical research council sum scale; CDAS = CIDP disease activity status.

a *t* test.

b MWU-Test.

Out of the 30 patients with CIDP, 7 patients had active disease and 23 patients had stable disease status according to CIDP disease activity status (CDAS) (24). To measure the level of muscle strength impairment, the Medical Research Council (MRC) sum scale (21, 22) was assessed. A sum score of 80 indicates normal muscle strength. The inflammatory neuropathy cause and treatment (INCAT) disability score (23) was used to assess the clinical disability in daily arm and leg mobility. INCAT score is inversely related to function, with 0 representing no functional impairment and 10 representing inability to make any purposeful movement with either arms or legs. The Rasch-built overall disability scale (R-ODS) is a linearly weighted scale and a patient-reported outcome measure that specifically captures activity and social participation limitations in patients with inflammatory neuropathies (20). It ranges from 0 (maximum disability) to 48 (no disability). Clinical scores and demographic data of CIDP patients and disease controls, as well as the subgroup analysis of patients with active and stable disease status as defined by CDAS, are summarized in Tables 1 and 2.

**Table 2:** Demographic and clinical data of the CIDP cohort with active or stable disease according to CDAS

Demographic Parameter	CDAS		*P*-value	Cohen’s d
	Active (n, 7)	Stable (n, 23)		
Sex [Male, %]	62.5%	46.9%	-	-
Age [y, mean ± SD]	61.9 ± 10	60.3 ± 13	0.751^a^	0.12
BMI [mean ± SD]	29 ± 3.7	28.4 ± 4.9	0.501^b^	0.13
INCAT [mean ± SD]	2.4 ± 1.4	2.8 ± 1.8	0.549^a^	−0.23
R-ODS [mean ± SD]	35.1 ± 10.4	34.1 ± 11.9	0.961^b^	0.09
MRC [mean ± SD]	55.7 ± 2.8	52.6 ± 7.1	0.416^b^	0.48

Statistical analysis in eAppendix 8.

CDAS, CIDP disease activity status; BMI, core; R-ODS, Rasch-built overall disability scale; MRC, medical research council sum scale.

a *t* test.

b MWU-Test.

### Shotgun lipidomic analysis reveals a significant difference in the lipid class of diacylglycerols (DAG)

**Fig. 1:**
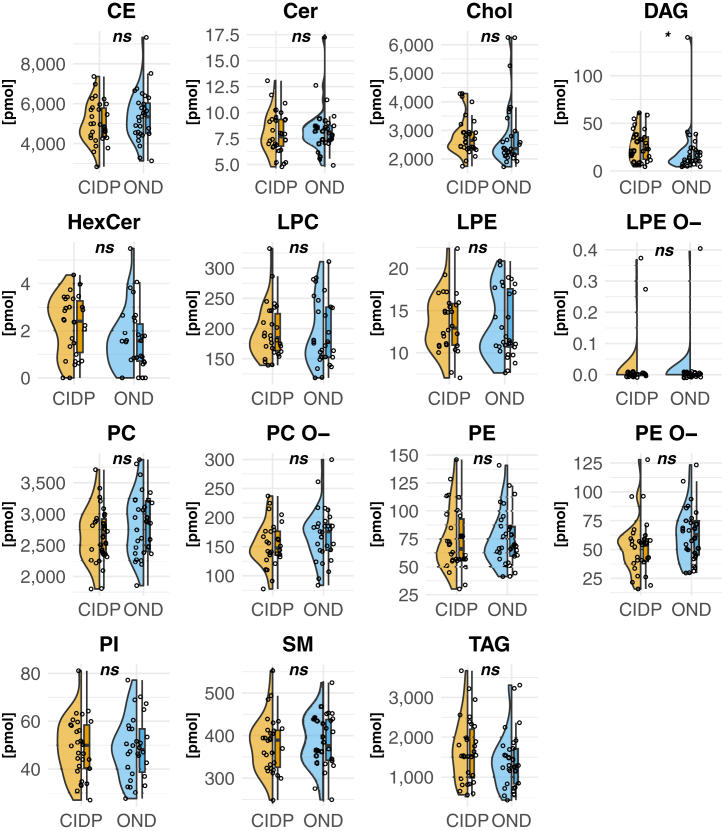
Class Comparison. Box-Plot with horizontal half violin Plot on the left side and individual values presented by dots. Visualizing the abundances of lipid classes in picomole [pmol]. Statistical analysis in eAppendix 9; n = 30 versus 30; *P* > 0.05 = ns; *P* < 0.05 = ∗. CE = Cholesteryl ester; Cer = Ceramide; Chol = Cholesterol; DAG = Diacylglycerol; HexCer = Hexosylceramide; LPC = Lysophosphatidylcholine; LPE = Lysophosphatidylethanolamine; LPE-O = Ether-linked Lysophosphatidylethanolamine; PC = Phosphatidylcholine; PC O- = Ether-linked Phosphatidylcholine; PE O- = Ether-linked Phosphatidylethanolamine; PE = Phosphatidylethanolamine; PI = Phosphatidylinositol; SM = Sphingomyelin; TAG = Triacylglycerol.

To identify lipid biomarker signatures of CIDP-patients, mass spectrometry-based shotgun lipidomics of 30 plasma samples of patients with CIDP was performed and compared to 30 patients with OND (eAppendix 1). The lipidomic platform identified and quantified 669 lipid molecular species in 15 lipid classes. Total class comparison revealed a significant increase of diacylglycerol (DAG) in the CIDP cohort, with no significant differences in the other lipid classes (Fig. 1 and eAppendix 9).

### Lipid subspecies analysis:

**Fig. 2:**
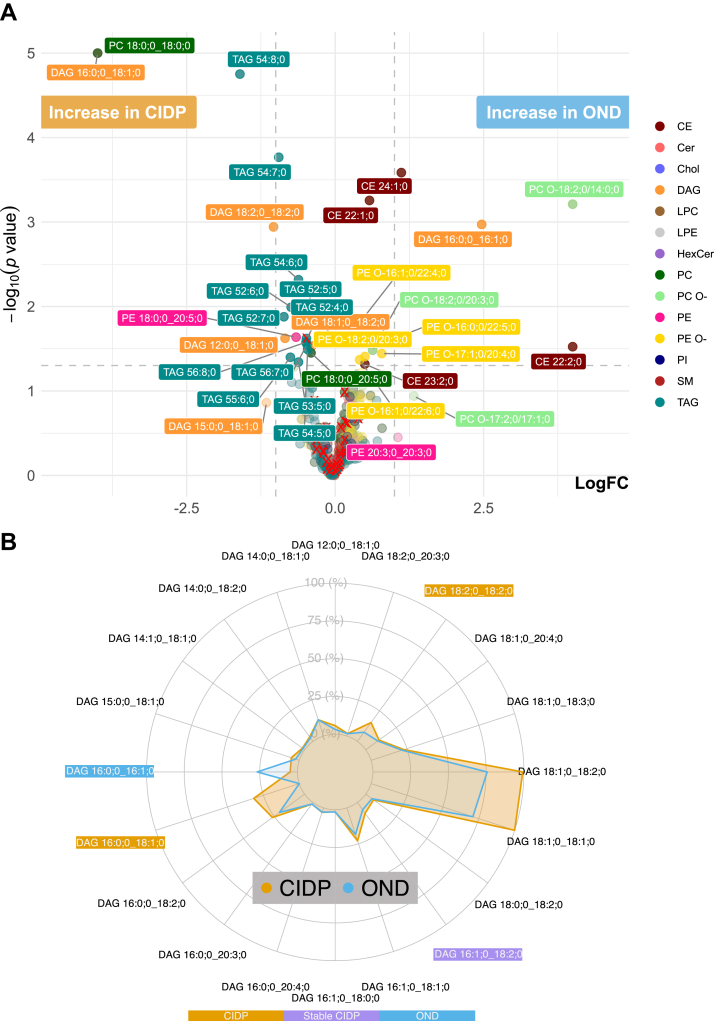
Volcano-Plot comparing CIDP- versus OND-Cohort and Radar-Plot of DAG. A: Volcano-Plot showing the differences in lipid subspecies in participants from the CIDP- versus OND-cohort. n = 30 versus 30. Colors represent lipid class classification. The horizontal dashed line indicates a *P*-value of 0.05 in the Welch’s *t* test. The vertical dashed line indicates a log2 fold change of 1. Only lipids with an absolute log2 fold change > 1 were annotated. Statistics in eAppendix 10. Lipids marked with red× have a high correlation with age and BMI (Supplemental Fig. 4 and eAppendix 4). B: Spider plot visualizing 20 lipids selected based on highest abundance and significant alterations. LogFC = Logarithmic fold change; CE = Cholesteryl ester; Cer = Ceramide; Chol = Cholesterol; DAG = Diacylglycerol; HexCer = Hexosylceramide; LPC = Lysophosphatidylcholine; LPE = Lysophosphatidylethanolamine; PC = Phosphatidylcholine; PC O- = Ether-linked Phosphatidylcholine; PE O- = Ether-linked Phosphatidylethanolamine; PE = Phosphatidylethanolamine; PI = Phosphatidylinositol; SM = Sphingomyelin; TAG = Triacylglycerol.

We compared the transformed abundances of lipid subspecies using a Welch’s *t* test (eAppendix 10 and 11). The results are displayed as a Volcano-Plot, comparing the CIDP cohort to the OND cohort (Fig. 2A). Lipids exhibiting a high correlation with age and BMI are marked with a red× and were excluded from further evaluation due to potential age and BMI dependency (Supplemental Fig. 4 and eAppendix 4). The comparison of CIDP versus OND revealed a significant increase of DAG 16:0;0_18:1;0, DAG 18:2;0_18:2;0, PC 18:0;0_18:0;0 and TAG 54:8;0 as well as a significant decrease of CE 22:2;0, CE 24:1;0, DAG 16:0;0_16:1;0 and PC O- 18:2;0/14:0;0 in the CIDP group (eAppendix 12). These findings were further supported by the application of a supervised machine learning algorithm (oPLS-DA), revealing high VIP (Variable Importance in Projection) values for several ether-linked phosphatidylcholine (PC O-), DAG, cholesteryl ester (CE) and phosphatidylcholine (PC) subspecies (Supplemental Fig. 6).

**Fig. 3:**
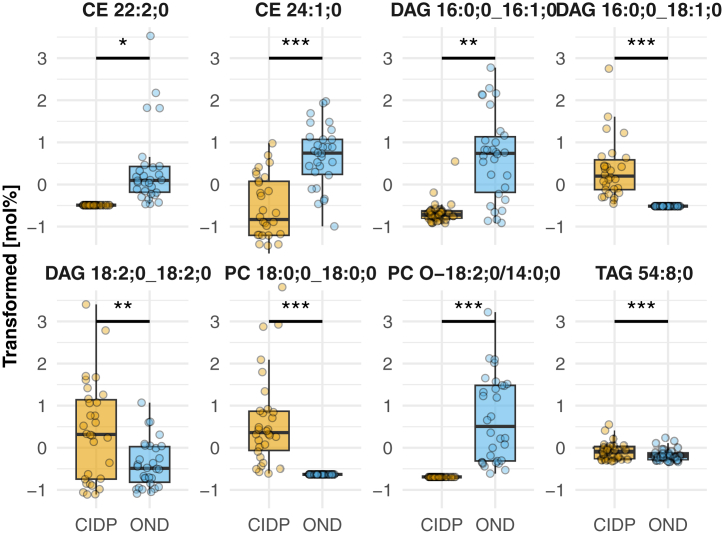
Significantly altered lipid species. Box-Plot with dots for individual values visualizing the significantly altered lipids by Fig. 2A (*P* value < 0.05 and |logFC| > 1)-eAppendix 12. CE = Cholesteryl ester; DAG = Diacylglycerol; PC = Phosphatidylcholine; PC O- = Ether-linked Phosphatidylcholine; TAG = Triacylglycerol.

To further assess the relevance of statistically significant changes in lipid levels, we compared these changes to the total composition of the corresponding lipid class. Therefore, we visualized the significantly altered lipids in the context of the top 20 most abundant lipids of the DAG class (Fig. 2B), illustrating the altered pattern of DAG species in CIDP patients. A detailed comparison of transformed abundances of the statistically relevant lipids (*P* value < 0.05 and |logFC| > 1) is presented in Fig. 3. The data underwent scaling and centering procedures to address the range of abundances, which resulted in the presence of negative values. Noteworthy, the shown CE and PC O- were decreased in patients with CIDP, while triacylglycerols (TAG) and PC were increased. To evaluate if our findings regarding DAG and TAG species are predominantly driven by imputed or low-abundance data we evaluated raw, unimputed, untransformed mol% values for all DAG and TAG lipids as shown in (Supplemental Fig. 9). Lipids, captured as significantly altered, were represented by both, high- and low-abundance lipid species within the TAG and DAG class (Supplemental Fig. 9). As indicated in Fig. 2A, an equivalent pattern applies to several lipid subspecies of the same class that are just below the significance level or do not reach a log2 fold change of 1. In the DAG class, changes in both directions were observed, with some subspecies being elevated and others being diminished in patients with CIDP, displaying a differential profile of DAG subspecies in CIDP patients.

### Correlation of clinical and lipidomic data:

**Fig. 4:**
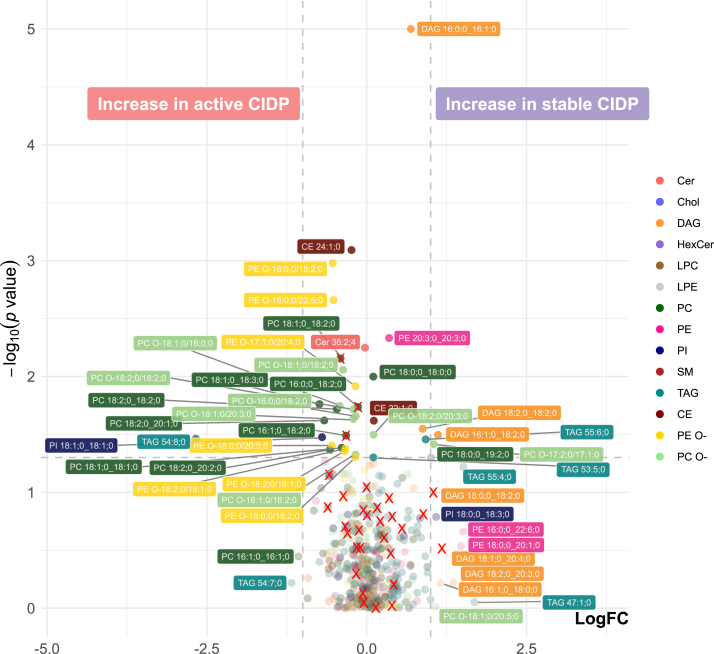
Volcano-Plot comparing active CIDP versus stable CIDP patients according to CDAS. Volcano-Plot showing the differences in lipid subspecies in participants from the active CIDP- versus stable CIDP-cohort. n = 30 versus 30. Colors represent lipid class classification. The horizontal dashed line indicates a *P*-value of 0.05 in the Welch’s *t* test. The vertical dashed line indicates a log2 fold change of 1. Only lipids with an absolute log2 fold change > 1 were annotated. Statistics in eAppendix 11. Lipids marked with red × have a high correlation with age and BMI (Supplemental Fig. 4 and eAppendix 4. Log FC = Logarithmic fold change, CE = Cholesteryl ester, Cer = Ceramide, Chol = Cholesterol, DAG = Diacylglycerol, HexCer = Hexosylceramide, LPC = Lysophosphatidylcholine, LPE = Lysophosphatidylethanolamine, PC = Phosphatidylcholine, PC O- = Ether-linked Phosphatidylcholine, PE O- = Ether-linked Phosphatidylethanolamine, PE = Phosphatidylethanolamine, PI = Phosphatidylinositol, SM = Sphingomyelin, TAG = Triacylglycerol.

To identify lipids that are associated with CIDP disease activity, we performed a subgroup analysis of the CIDP cohort and categorized according to CDAS into unstable active disease status (“active CIDP”) and stable disease status (“stable CIDP”). The results are visualized as volcano plot (Fig. 4). Lipids inhibiting a high correlation with age and BMI are marked with a red ×. We detected a significant increase of TAG 54:8;0 in patients with active CIDP, which also was increased in the general CIDP cohort versus the OND cohort. Further significantly altered lipids with a log2 fold change > 1 were *DAG 16:1;0_18:2;0* and *PC O-17:2;0/17:1;0*. Including lipids with a lower log2 fold change, a significant increase of several PC, PC O- and PE O- subspecies was observed in patients with active CIDP disease status, while all illustrated DAG subspecies were decreased in patients with active CIDP.

**Fig. 5:**
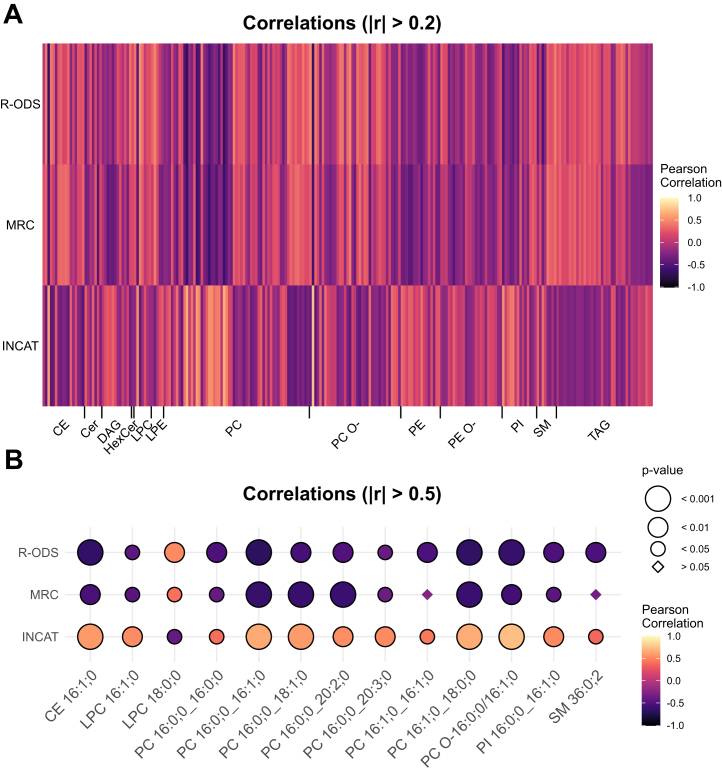
Correlation analysis. Heatmap with color coding by Pearson correlation displaying all 247 lipids with an absolute correlation r > 0.2 (A) and 21 lipids with an absolute r > 0.5 (B). p < 0.05 = ∗; *P* < 0.01 = ∗∗; *P* < 0.001 = ∗∗∗. Corresponding statistics in eAppendix 6. CE = Cholesteryl ester; Cer = Ceramide; DAG = Diacylglycerol; HexCer = Hexosylceramide; LPC = Lysophosphatidylcholine; LPE = Lysophosphatidylethanolamine; PC = Phosphatidylcholine; PC O- = Ether-linked Phosphatidylcholine; PE O- = Ether-linked Phosphatidylethanolamine; PE = Phosphatidylethanolamine; PI = Phosphatidylinositol; SM = Sphingomyelin; TAG = Triacylglycerol.

Next, we examined whether there were correlations between the detected lipids and clinical scores such as R-ODS, MRC, and INCAT (Fig. 5) using Pearson’s correlation. We discovered a set of lipids that decreased in concentration with increasing R-ODS and MRC scores, while increasing with higher INCAT scores, and vice versa (Fig. 5A). Noteworthy, R-ODS and MRC scores decrease with the severity of the disease, while INCAT score increases with functional impairment. Thus, explaining the reverse correlation in nearly all displayed lipids. Individual lipids from CE, lysophosphatidylcholine (LPC), PC, PC O-, phosphatidylinositol (PI), and sphingomyelin (SM) subspecies exhibited the strongest correlation with an absolute r > 0.5 (Fig. 5B). These lipids predominantly increased with higher disability in clinical scores. To evaluate the possibility of BMI and age acting as confounders, we included a correlation analysis for these values (Supplemental Fig. 7). Some lipids correlating with functional impairment in clinical scores also showed moderate correlation to BMI, while correlation with age was inferior.

## Discussion

Biomarkers for CIDP are urgently needed in clinical practice since the disease is frequently incorrectly diagnosed (31) or inappropriately monitored and treated (8). Effective and partially cost-intensive treatment options are available for most of these patients, but there is an unmet clinical need for objective biomarkers to improve diagnosis as well as assess disease activity and progression reliably to allow for individualized therapeutic decisions in patients. Considering the underlying pathophysiological processes such as inflammation, demyelination and axonal damage, several studies have attempted to find potential blood biomarkers for assisting the diagnosis and evaluating the activity of CIDP. Due to the heterogeneity of the disease with different clinical courses and the multifaceted pathophysiology, a set of differing biomarkers was considered useful to depict the diverse aspects of the disease (32). The plasma lipidome has been shown to reflect systemic inflammatory processes and has the potential to indicate neuropathological processes such as axonal damage or demyelination (15, 33, 34). Thus, we speculate that plasma lipid profiles could reflect various pathophysiological aspects of the disease. This exploratory study aimed to assess whether plasma lipid profiles could reflect CIDP-related pathological processes and support future biomarker development.

A noteworthy limitation of this study is the absence of a healthy control group. While including such a group would have enabled clearer identification of disease-specific alterations, this was not possible due to the significant age disparity typically observed between healthy individuals and the predominantly older CIDP population. Furthermore, our primary objective was to evaluate lipidomic patterns with direct clinical relevance, prompting us to compare patients having CIDP with individuals suffering from other non-demyelinating neurological disorders (OND). This comparator group reflects a more clinically relevant scenario, in which distinguishing CIDP from other neurological conditions can be challenging from a diagnostic perspective. However, this design limits our ability to differentiate disease-specific changes from general alterations associated with neurological illness. Future studies, including age- and sex-matched healthy controls, are needed to further delineate CIDP-specific lipidomic signatures.

In the present study, we performed a broad-spectrum lipidomic analysis of plasma samples of a well-defined group of patients with CIDP. We specified disease activity status and functional impairment using several well-established clinical scores. Thus, we were able to detect a set of lipids correlating with CIDP-diagnosis, including CE, DAG, TAG, PC and PC O- species. Additionally, we performed a subgroup analysis identifying lipid species related to CIDP disease activity status, with TAG 45:8;0, DAG 16:1;0_18:2;0 and PC O-17:2;0/17:1;0 being significantly altered lipids. By correlating our lipidomic analysis to several clinical disability scores, we found a lipidomic pattern that was associated with increased disability status in all tested scores.

A general pitfall in the evaluation of lipidomic datasets involves missing values due to measurements falling below the limit of detection (LOD), necessitating imputation strategies that potentially result in bias. In this study, we applied a structured approach to minimize such effects and to maintain the interpretability of lipidomic differences observed in patients with CIDP as described in the methods section. Additionally, lipids showing strong correlations with potential confounders such as age or BMI were excluded, and a fold-change threshold (|log_2_ FC| > 1) was applied to emphasize biologically relevant differences. To account for the limitations of untargeted approaches, these steps were implemented to minimize imputation bias and enhance the robustness of the results. Further targeted studies will be needed to confirm the observed lipid alterations in larger cohorts, with a more detailed assessment of potential confounding factors such as the sex of patients and therapeutic interventions.

Several of the lipid species identified as being significantly altered in CIDP had relatively low detection frequencies across the cohort, particularly in the OND group. This is a known limitation of untargeted shotgun lipidomics, especially when detecting low-abundance lipids near the limit of detection. Although we applied a structured imputation and filtering strategy to reduce potential bias, the resulting conclusions regarding low-abundance lipid species should be interpreted with caution. Quantifying DAG species presents particular challenges in this regard due to analytical issues such as poor ionization and low abundance compared to other neutral lipids, such as TAGs and CEs (35, 36). Future studies using targeted, high-sensitivity lipidomic approaches are warranted to validate these candidate lipids in independent and larger datasets.

### Diagnose-related lipid patterns

Demyelinating polyradiculoneuropathy is a pathological hallmark in patients affected by CIDP and AIDP. While lipidomic analyses in AIDP are limited, a few studies have reported alterations in lipid metabolism (16, 17, 37). In the CSF of patients with Guillain-Barré Syndrome (GBS), an increase in DAG has been observed compared to controls with non-demyelinating diseases (17). This finding aligns with our results, where we detected a significant elevation in the overall DAG class in CIDP patient plasma. Similarly, in CNS demyelinating diseases, such as multiple sclerosis (MS), DAG levels in plasma were found to be elevated regardless of disease course (38).

The consistent increase in the DAG class across different demyelinating diseases suggests a potential association with inflammatory myelin damage. DAG production may be influenced by lipoprotein lipase (LPL) activation or phospholipase C (PLC) activity (17). LPL upregulation has been implicated in the acute response to myelin degradation, facilitating lipid scavenging and reutilization in degenerating peripheral nerves, with macrophages playing a key role in this process (39). PLC, on the other hand, can be activated by inflammatory cytokines, leading to the hydrolysis of phosphatidylinositol 4,5-bisphosphate (PIP2) into inositol trisphosphate (IP3) and DAG (40), potentially increasing DAG production at sites of inflammation and demyelination. Additionally, the breakdown of the lipid-rich myelin sheath may release various lipid-derived molecules, contributing to an elevated turnover of DAG species.

However, an alternative explanation for the observed DAG increase relates to global changes in plasma lipid metabolism in the context of inflammatory processes (41). DAG can be generated through the hydrolysis of TAG by lipases (42) and alterations of this interplay might reflect systemic inflammation (43). Additionally, there is rising evidence that DAG levels may be influenced by the metabolism of TAG-rich lipoproteins, including HDL and LDL fractions, providing a potential explanation for the increased levels of both TAG and DAG species in our dataset, correlated with the CIDP diagnosis.

Functionally, DAG species act as key second messengers in intracellular signaling pathways, particularly through the activation of protein kinase C (PKC) (41, 42). In the context of demyelination, aberrant DAG-PKC signaling may contribute to immune activation, promoting the recruitment of T cells and macrophages that target the myelin sheath (44, 45, 46, 47). Furthermore, DAG-PKC signaling can induce oxidative stress by increasing reactive oxygen species (ROS) production (48), potentially leading to damage of myelin-producing cells (49, 50). In the CNS, dysregulated PKC signaling has also been implicated in oligodendrocyte proliferation and differentiation (51, 52, 53).

Despite the overall increase in the DAG class observed in our study, some DAG subspecies exhibited bidirectional alterations. Similar findings were reported in previous lipidomic analyses of MS, where differential DAG subspecies contributed to altered class composition (38). These results highlight the complexity of DAG metabolism and suggest that specific DAG species, rather than total DAG levels, may play distinct roles in disease mechanisms.

TAG lipids are primarily recognized for their role in energy storage, but their involvement in demyelinating diseases may be linked to broader disruptions in lipid metabolism and inflammation. Elevated TAG levels and TAG-rich lipoproteins have been shown to enhance inflammatory responses in macrophages, monocytes, and T-cells (54, 55, 56). In line with our findings, increased TAG species have been reported in both the CSF and serum of GBS patients (17, 57). Similarly, diabetic neuropathy has been associated with significantly elevated TAG levels (58), with higher TAG concentrations correlating with an increased risk of neuropathy in diabetes mellitus type 1 (DM1) and DM2 patients (59, 60). Moreover, fibrates—lipid-lowering agents that reduce TAG and increase HDL—have shown potential benefits in diabetic neuropathy (61), though their impact on GBS and CIDP remains unclear.

The observation of elevated DAG levels in the plasma lipidome of patients with CIDP, could be potentially linked to a trend towards elevated TAG concentrations. Given that DAG can arise from lipase-mediated TAG hydrolysis (62), these changes may reflect broader alterations in plasma lipid metabolism. It was demonstrated that TAG-rich lipoproteins play a central role in systemic inflammatory conditions (63), including atherosclerosis and rheumatoid arthritis, influencing both lipid transport and immune modulation. While our current dataset does not specifically analyze the lipid compositions of TAG-rich lipoproteins, future studies incorporating direct assessments of lipoprotein subfractions might add to the current knowledge of plasma lipid alteration in neurological diseases (64).

Future research should aim to validate these findings in larger cohorts to better understand the interplay between lipid metabolism and CIDP pathophysiology and assess the potential of DAG and TAG levels to be employed as biomarkers in patients with CIDP.

Focusing exclusively on CIDP, previous studies have primarily investigated sphingomyelin (SM) as a potential lipid biomarker of the demyelinating process (65). A fluorescence-based assay demonstrated elevated SM levels in the cerebrospinal fluid (CSF) of CIDP patients compared to individuals with non-demyelinating neurological diseases, with even higher SM levels observed in active CIDP compared to both stable CIDP and controls. In contrast, our plasma lipidomic analysis using mass spectrometry did not reveal significant differences in the total SM class or its subspecies when comparing CIDP patients with controls. However, we did observe a notable correlation between specific SM species, such as SM 36:0;2, and increased clinical disability scores.

The observed discrepancy between CSF and plasma findings highlights the challenges in using peripheral lipidomic changes to infer localized pathological processes in CIDP. While CSF is in immediate contact with the site of inflammation and demyelination in polyradiculitis, its clinical use is limited due to the risks associated with lumbar puncture. Plasma, on the other hand, is more readily accessible, suitable for longitudinal assessment and may reflect systemic metabolic alterations related to disease activity.

Future studies integrating both CSF and plasma lipidomic analyses, along with longitudinal disease monitoring, may provide further insights into the metabolic changes underlying CIDP and their potential utility for clinical decision-making.

Glycosphingolipids, particularly galactosylceramide and sulfatide, are major constituents of myelin. In our CIDP cohort, no increase in glycosphingolipids was observed compared to patients with other neurological diseases. While hexosylceramides (HexCer) - encompassing galactosylceramide and glucosylceramide - were detected, our current analytical method could not differentiate between these species, and sulfatides were not assessed due to low abundance in the untargeted approach. The lack of significant changes in plasma HexCer may stem from rapid degradation of myelin lipids post-demyelination and efficient clearance by phagocytic cells, limiting their detectability. Future targeted lipidomic studies might help to assess whether specific myelin lipid components released during demyelination can be detected in body fluids such as plasma or CSF.

Other lipid subspecies that were significantly altered in patients with CIDP in our study comprised of subsets of Cholesterol esters (CE), PC O-, TAG, and PC species.

Notably, plasma CE levels were reduced in patients with CIDP. As storage molecules of cholesterol, CE play a crucial role in membrane integrity, protein trafficking, and myelin compaction in Schwann cells (66, 67).

Findings from previous studies suggest that cholesterol metabolism may differ between acute and chronic demyelination. It has been hypothesized that during acute-phase remyelination, as seen in rapidly demyelinating lesions, cholesterol is primarily recycled from myelin debris, with oligodendrocytes and astrocytes downregulating cholesterol synthesis-related transcripts. In contrast, chronic demyelination may require increased expression of sterol metabolism genes to sustain remyelination (68). Consistent with this, lower plasma cholesterol levels have been reported in acute demyelinating diseases such as Guillain-Barré syndrome (GBS) and multiple sclerosis (MS) (37).

Given that CIDP is a chronic disease with repeated cycles of demyelination and remyelination, the observed reduction in CE levels may reflect an increased demand for cholesterol to support ongoing myelin repair. However, whether this aligns with the hypothesized metabolic differences between acute and chronic demyelination remains to be clarified in future studies.

In patients with CIDP, we observed a decrease in several ether-linked phospholipid subspecies, including PC O- and PE O-, while some PC species were elevated. Ether phospholipids, characterized by an ether bond at the*sn-1* position of the glycerol backbone, are essential components of cellular membranes, including the myelin sheath. Compared to their ester-linked counterparts, they offer increased resistance to oxidative stress and contribute to membrane stability (69, 70, 71).

A reduction in ether phospholipids, particularly plasmalogens, has been linked to increased susceptibility of myelin to oxidative damage, a key feature of demyelination. Studies in plasmalogen-deficient mice demonstrated heightened vulnerability of sciatic nerve myelin to reactive oxygen species (ROS) (72). Similar decreases in ether lipids have been reported in neurodegenerative diseases, including Alzheimer’s and Parkinson’s disease (73, 74, 75), as well as in multiple sclerosis (38, 76), suggesting a broader role in neuroprotection and myelin integrity. Furthermore, the importance of ether phospholipids in Schwann cell development and differentiation has been demonstrated in a mouse model of Rhizomelic chondrodysplasia punctata (77).

### Lipid patterns associated with CIDP disease activity

Among others, the detection of serum autoantibodies, as well as serum and CSF complement and cytokine profiles, have been suggested as biomarkers of the inflammatory process of CIDP pathogenesis (78). In particular, autoantibodies against different gangliosides, neurofascin isoforms (NF155 and NF140/186), Contactin 1, and CASPR1 have been described in approximately 10% of CIDP patients and could be helpful to distinguish different CIDP subtypes or (para-) nodopathies (79, 80, 81) as well as potentially assist disease activity monitoring (82). Another potential biomarker that is involved in the signaling pathway of inflammatory processes is serum calprotectin (CLP). CLP was shown to be significantly associated with active disease course according to CDAS (83). Since persistent disease activity is likely to be associated with ongoing inflammatory activity, we also used the CDAS to stratify disease activity. However, due to the heterogeneity of the disease, varying therapeutic concepts and the limited sample size, only a few lipids with a large and significant difference between the two groups were detected. We observed a significant increase of TAG 54:8;0, a TAG species we also found to be altered in CIDP patients in general as compared to OND. Additionally, other significantly altered lipids with a log2 fold change > 1 were DAG 16:1;0_18:2;0 and PC O-17:2;0/17:1;0. When considering lipids differing amongst active and stable CIDP with less pronounced fold changes, members of the phospholipid classes PC, PC O- and PE O- appeared to be altered. Phospholipids could play diverse roles in the inflammatory processes, acting as structural components of cell membranes, signaling molecules, and modulators of immune responses. As an example, PC is involved in the formation of lipid rafts, which are specialized microdomains that play a key role in cell signaling and the regulation of immune responses (84). In addition, plasmalogens, a subclass of PC O-, are precursors to bioactive lipid mediators that can modulate inflammatory processes (85, 86, 87).

In contrast, several DAG subspecies appeared to be decreased in patients with active CIDP, again, indicating the broad and diverse functionality of DAG species during active inflammatory processes as well as their involvement in membrane synthesis in the context of reparative mechanisms as discussed above.

In general, we hypothesize that lipidomic analysis regarding neurological diseases should also consider lipid species pattern evaluation and correlation with clinical features. Prospective longitudinal trials and systematic testing from the point of diagnosis through the course of therapeutic interventions would be highly beneficial and necessary for identifying and validating biomarkers that can distinguish active from stable CIDP. This pilot trial and the data regarding active disease status might point towards the usability of lipidomic analyses in this context.

### Lipidomic patterns correlated with clinical deterioration

In the literature, neurofilament light chain (NfL) (45) and peripherin (88) have been proposed as markers of axonal damage in the context of CIDP. Beyond the extent of demyelination, axonal damage resulting from the inflammatory, demyelinating process is likely to be an additional driver of clinical disability (89), a sign of insufficient clinical treatment and can be observed and confirmed, for example, by electrophysiological assessment as well as by using clinical scores.

Correlating lipid profiles to the functional clinical scores R-ODS, MRC, INCAT, we found a set of lipids strongly correlating with clinical disability. Remarkably, lipids, that were decreased or elevated in patients with lower R-ODS and MRC scores were inversely correlated with INCAT score, consistent with the contrasting scaling of the scores. Since these well-established clinical scores might also be influenced by other variables, such as age and BMI, the results must be interpreted with caution. Nevertheless, we observed a strong correlation regarding several lipids (including some CE, LPC, PC, PC O-, PI, and SM species), which showed only a weak correlation with age and BMI (Fig. 5). In GBS patients, a set of PC and SM was described that was inversely correlated with the Hughes functional grading scale (37). Analogous to these findings, our set of markers that correlated with functional scores also included some PC and SM species that were inversely correlated with R-ODS and MRC score and positively correlated with INCAT score, suggesting an increase in these lipid species in clinically worse patients.

Given that CIDP is an immune-mediated demyelinating disease, changes in the plasma lipidome might reflect multifaceted processes, including demyelination, inflammation and axonal damage, and could possibly be used not only as diagnostic blood biomarkers, but also to indicate disease activity and underlying pathophysiological processes.

Taken together, we identified distinct lipid signatures associated with CIDP diagnosis as well as CIDP disease activity and clinical disability. Our findings might contribute to a better understanding of the pathophysiology of the disease and the discovery of clinically relevant biomarker signatures. Further studies are needed and should include prospective longitudinal designs, larger cohorts, and healthy control-groups. Additionally, a combination of multiple biomarkers could be useful to enhance the accuracy in distinguishing active from stable CIDP. These studies are essential for advancing the understanding of CIDP, optimizing treatment strategies, and ultimately improving patient outcomes by enabling more precise, personalized care.

## Data availability

Data not presented within this article is accessible in an anonymized format upon request by reaching out to the corresponding author.

## Supplemental data

This article contains supplemental data.

## Conflict of interest

The authors declare the following financial interests/personal relationships which may be considered as potential competing interests:

K. a. d. B, S. S. J. L., L. -M. B., J. Z., M. N., L.V. and D. F. declare no conflict of interest. C. K. is employee and shareholder of Lipotype GmbH, Dresden.

## Supplemental data

Supplementary Figures

eAppendix
