# Supplementary material for: Plasma lipidomic patterns associated with disease activity in chronic inflammatory demyelinating polyradiculoneuropathy (LIPID-CIDP)
Source: J Lipid Res. 2025 Sep 17;66(11):100903. doi: 10.1016/j.jlr.2025.100903 (PMC12593536; doi:10.1016/j.jlr.2025.100903)
Supplement: Supplementary Figures [file mmc1.docx]

# eFigures

**Plasma Lipidomic Patterns Associated with Disease Activity in Chronic Inflammatory Demyelinating Polyradiculoneuropathy: A Case-Control Study (LIPID-CIDP)**

Kristina auf dem Brinke^1^, Lisa-Marie Borsch^1^, Christian Klose^2^, Jana Zschüntzsch^1,3^, Liza Vinhoven^4^, Manuel Nietert^4^, Seyed Siyawasch Justus Lattau^1†^, Dirk Fitzner^1†*^

^1^: Department of Neurology, University of Göttingen, 37075 Göttingen, Germany

^2^: Lipotype GmbH, 01307 Dresden, Germany

^3^: Neuromuscular Center Göttingen, Department of Neurology, University of Göttingen, 37075 Göttingen, Germany

^4^: Department of Medical Bioinformatics, University of Göttingen, 37075 Göttingen, Germany

^†^ These authors jointly supervised this work

^*^ Author to whom correspondence should be addressed.

Correspondence:

Dr. med. S. S. J. Lattau

Department of Neurology, University of Göttingen, Germany

+49 551 3966650

Email: [justus.lattau@med.uni-goettingen.de](mailto:justus.lattau@med.uni-goettingen.de)

PD Dr. med. D. Fitzner

Department of Neurology, University of Göttingen, Germany

Phone: +49 551 3967087

Email: [d.fitzner@med.uni-goettingen.de](mailto:d.fitzner@med.uni-goettingen.de)

[eFigure 1 Cleaning. 2](#_Toc181880140)

[eFigure 2 Imputation. 3](#_Toc181880141)

[eFigure 3 Transformation. 4](#_Toc181880142)

[eFigure 4 Age and BMI correlation. 5](#_Toc181880143)

[eFigure 5 Principal Component Analysis. 6](#_Toc181880144)

[eFigure 6 oPLS-DA. 7](#_Toc181880145)

[eFigure 7 Correlation Analysis. 8](#_Toc181880146)

[eFigure 8 Correlation of selected lipids. 9](#_Toc181880147)

eFigure 9 Raw mol % abundances of DAG (top) and TAG (bottom) species in CIDP versus OND………10


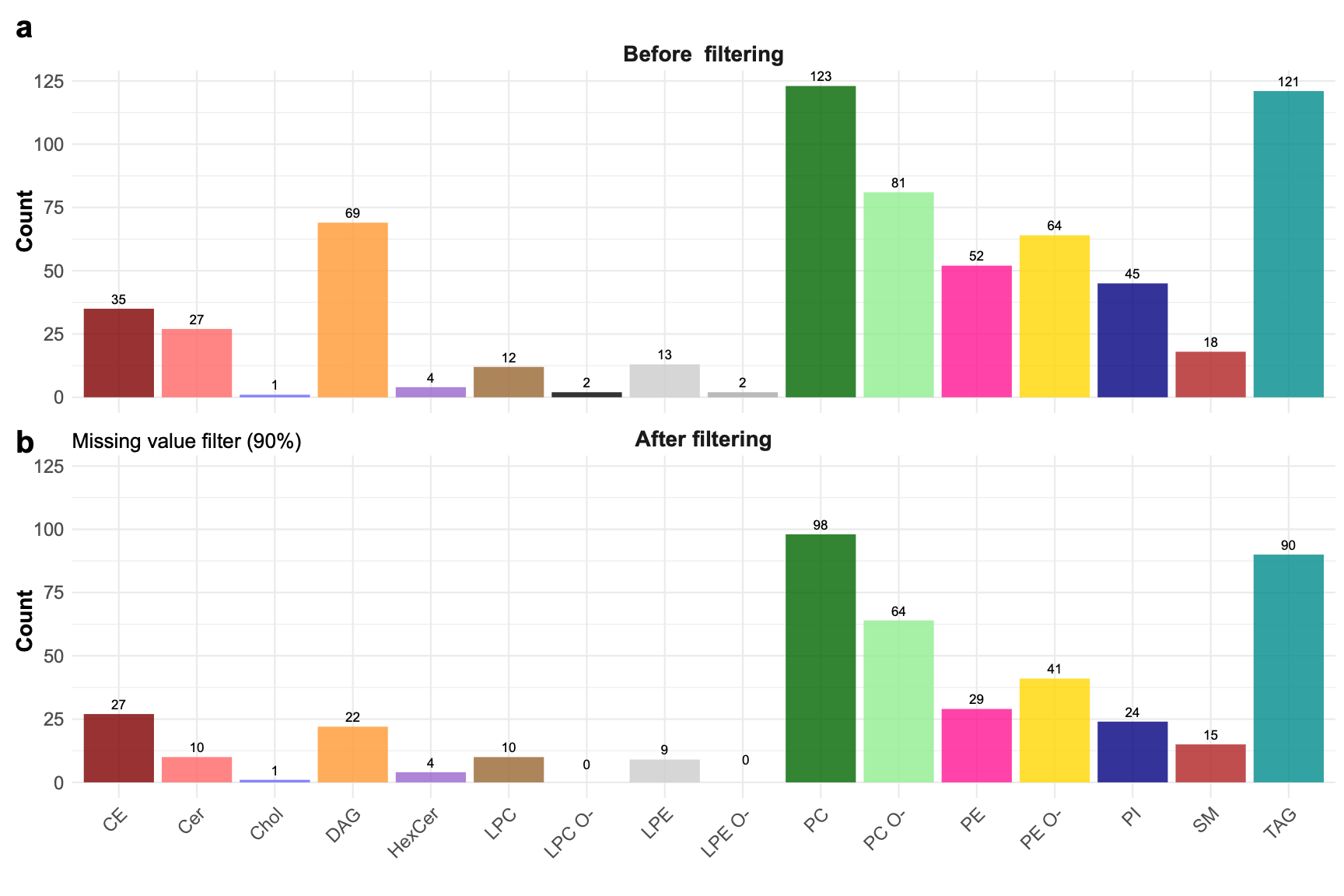


eFigure 1 Cleaning.

Illustration of the number of lipid species within each class before (**a**) and after (**b**) applying a missing value filter of 90%. Each bar represents the number of lipid species within the respective class, with distinct colors used for each class for clear differentiation. The total number of lipid species before filtering is 669, and after filtering is 444. The application of the missing value filter significantly reduces the number of lipid species in most classes.


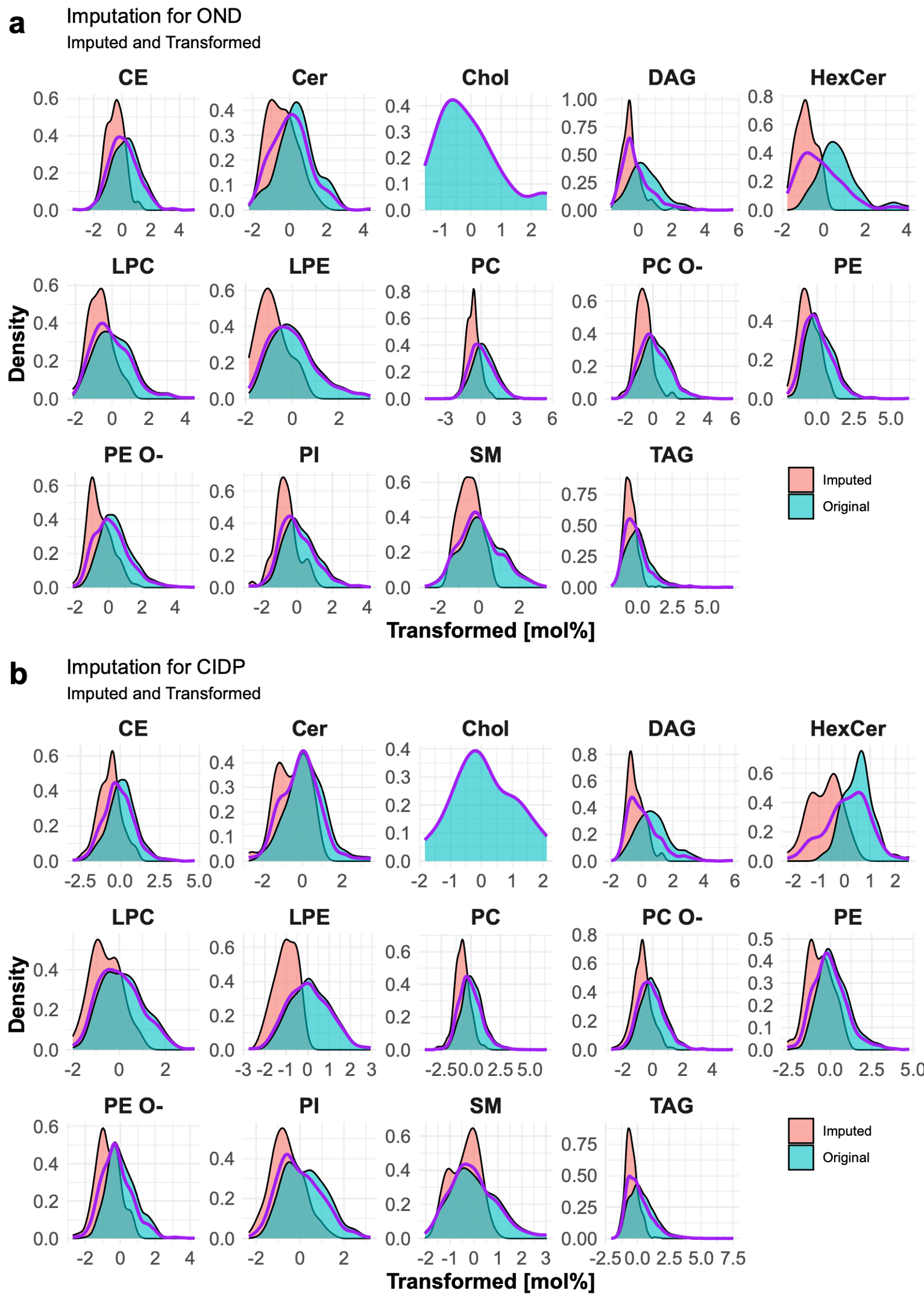


eFigure 2 Imputation.

Density-Plots for each lipid class, grouped by OND-cohort (**a**) and CIDP-cohort (**b**), visualizing the original (blue), imputed (red), and combined data (purple).

CE = Cholesteryl ester, Cer = Ceramide, Chol = Cholesterol, DAG = Diacylglycerol, HexCer = Hexosylceramide, LPC = Lysophosphatidylcholine, LPE = Lysophosphatidylethanolamine, PC = Phosphatidylcholine, PC O- = Ether-linked Phosphatidylcholine, PE O- = Ether-linked Phosphatidylethanolamine, PE = Phosphatidylethanolamine, PI = Phosphatidylinositol, SM = Sphingomyelin, TAG = Triacylglycerol.


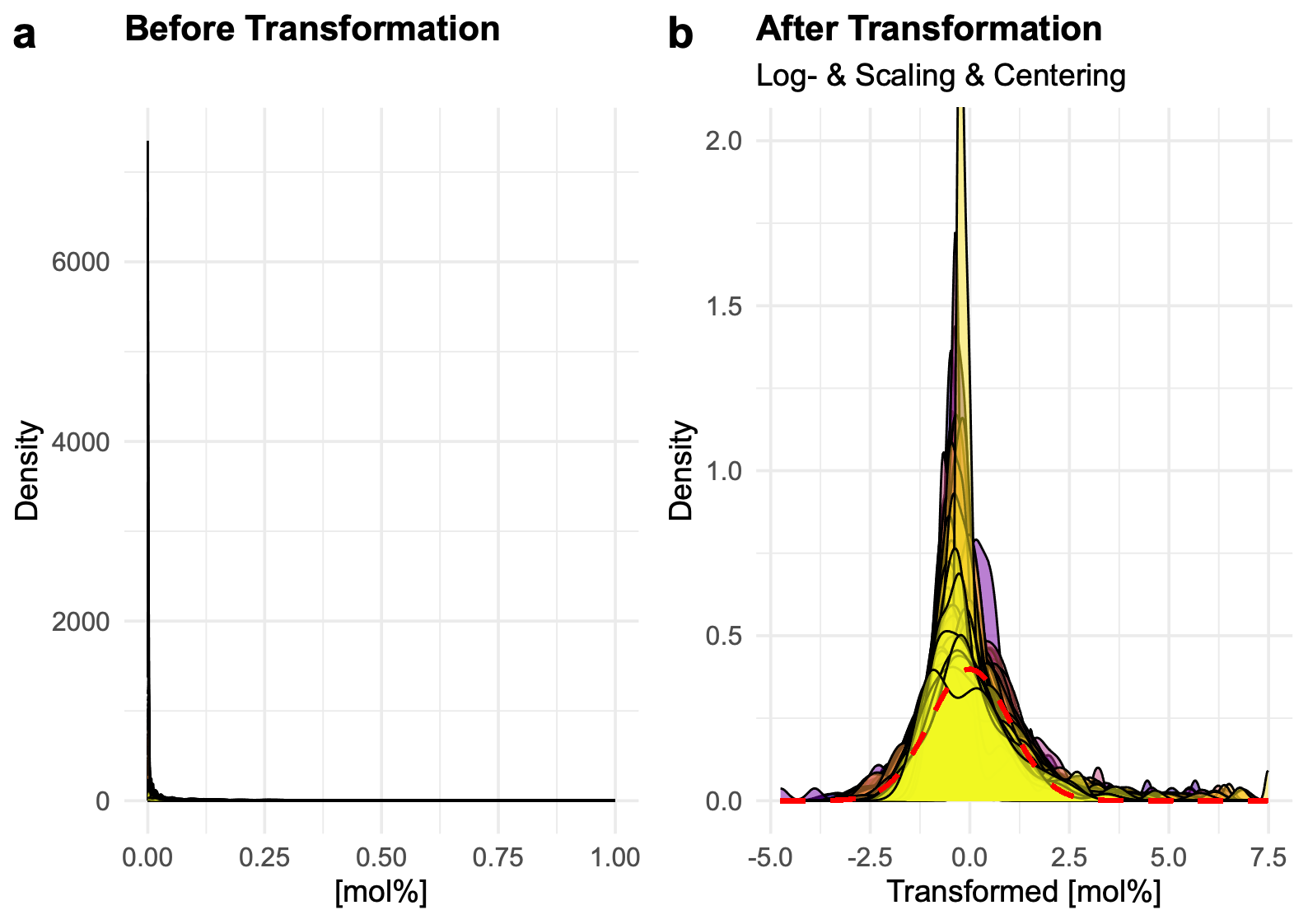


eFigure 3 Transformation.

Density-plot of lipid species before (**a**) and after applying log scaling (divides each lipid measurement species by its standard deviation) and centering (subtracts the mean value from each lipid measurement) transformations (**b**). Each color represents a different lipid species. The dotted red line indicates a Gaussian normal distribution. By applying these transformations, the data becomes approximately normally distributed, which allows for the application of parametric statistical tests.


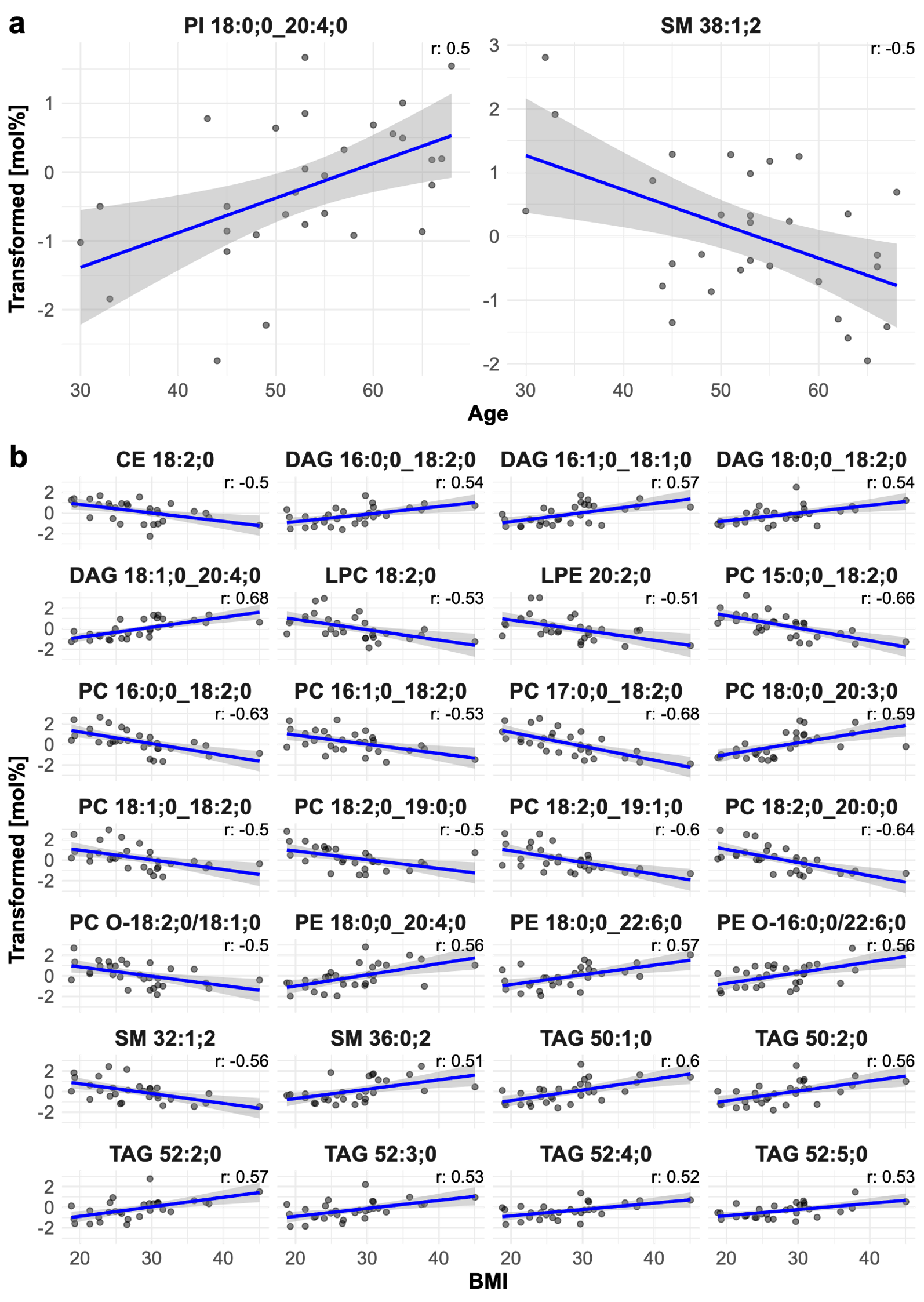


eFigure 4 Age and BMI correlation.

Visualization of lipids with an absolute Pearson correlation r > 0.5. Identifying these lipids as potentially relevant correlated with age (**a**) and BMI (**b**).


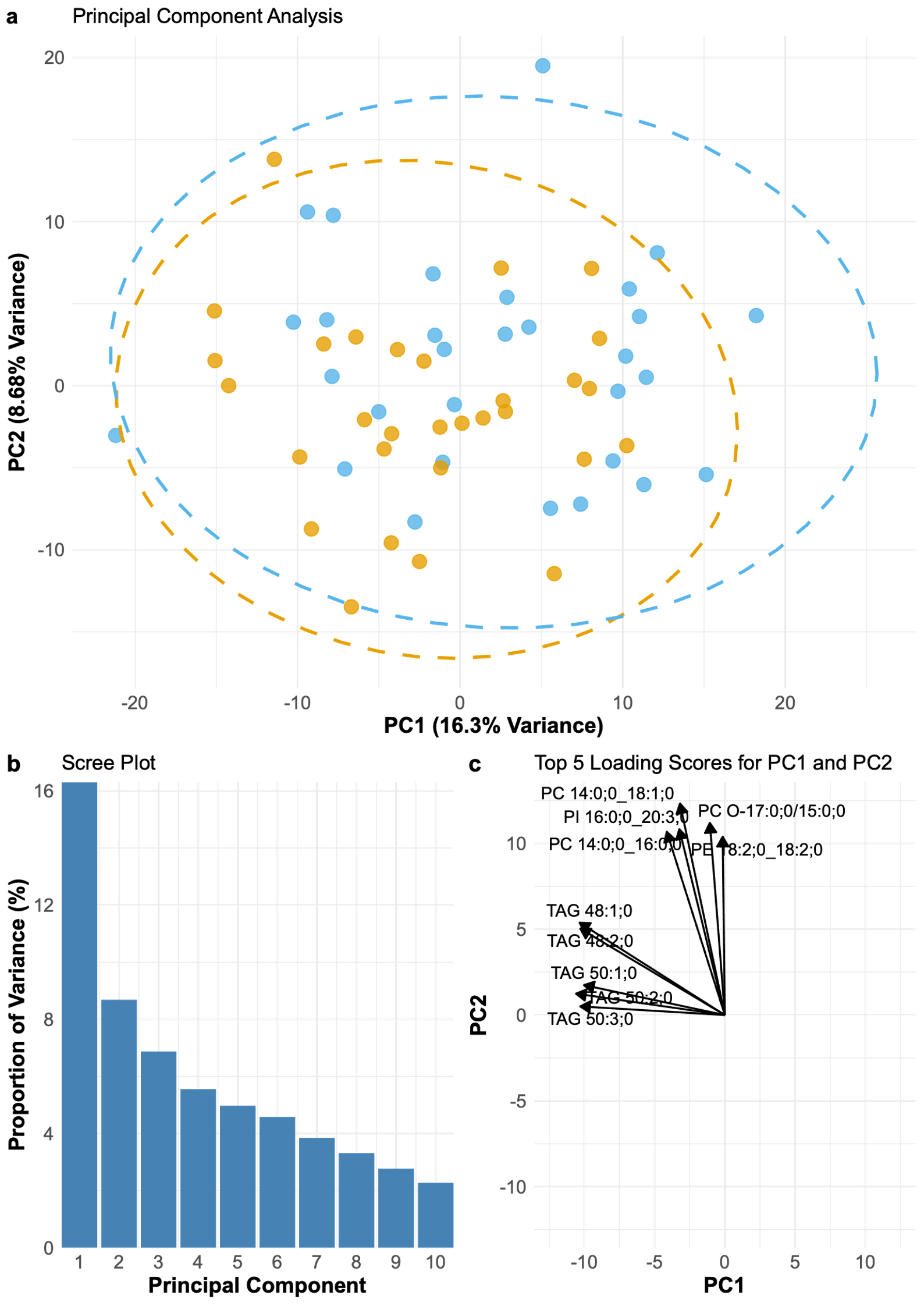


eFigure 5 Principal Component Analysis.

Principal Component Analysis (PCA) of all 444 lipids, grouped by the CIDP- cohort and OND-cohort (**a**). Scree plot (**b**) and a loading plot (**c**) for the top 5 important lipids of Principal Component 1 (PC1) and Principal Component 2 (PC2).


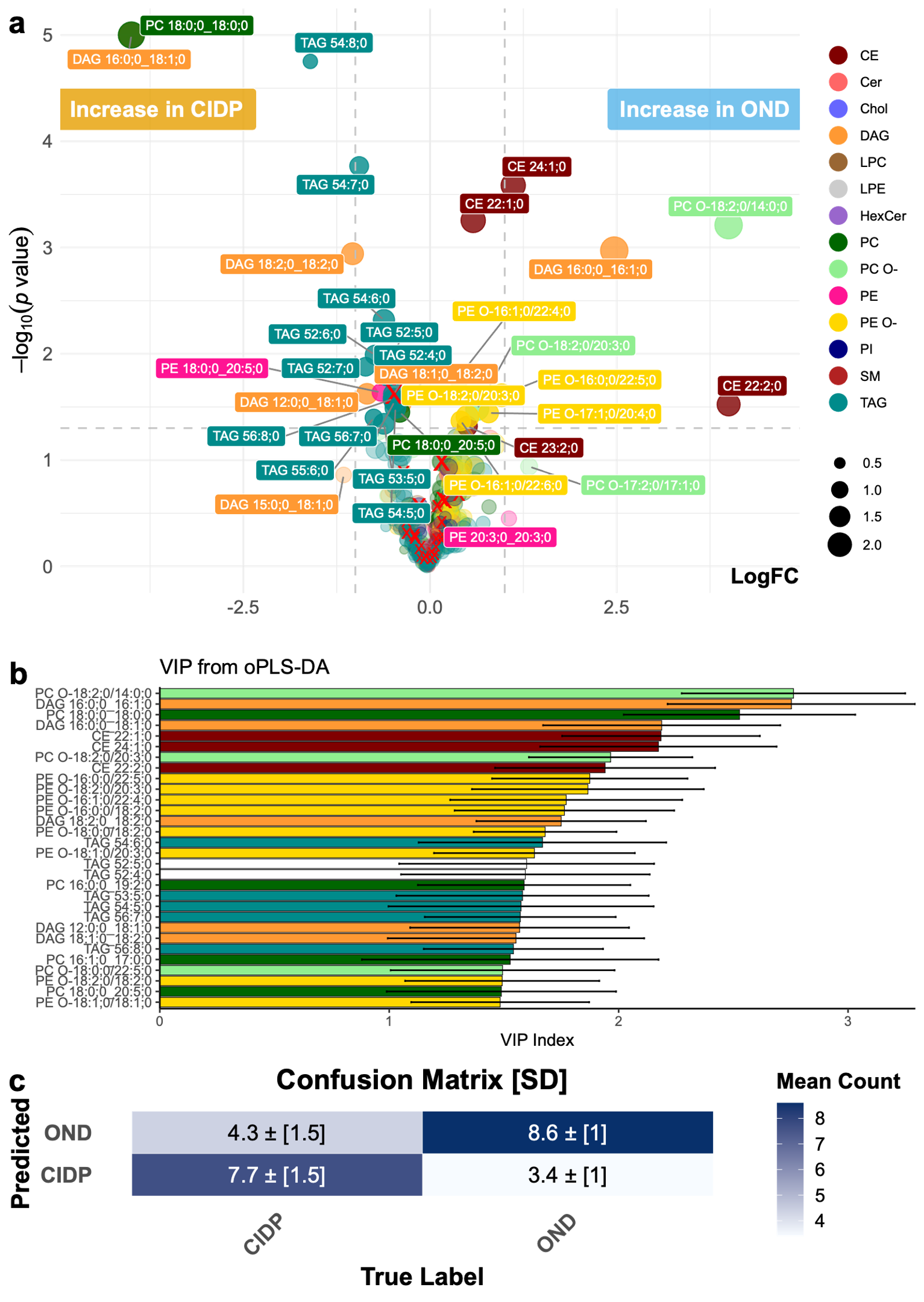


eFigure 6 oPLS-DA.

Volcano-Plot comparing the CIDP cohort versus the OND cohort, with the size of the dots adjusted by the VIP score from an oPLS-DA (orthogonal Partial Least Squares Discriminant Analysis) comparing CIDP-cohort vs. OND-cohort (**a**). The red 'x' marks indicate lipids with a relevant correlation to age and BMI. VIP scores from the oPLS-DA analysis, highlighting the most important variables in differentiating between the CIDP and OND cohorts (**b**). Classification accuracy of the oPLS-DA model when tested on a separate testing dataset, which comprises 40% of the total dataset (**c**). Additional statics in supplementary file 11.

LogFC = Logarithmic fold change, CE = Cholesteryl ester, Cer = Ceramide, Chol = Cholesterol, DAG = Diacylglycerol, HexCer = Hexosylceramide, LPC = Lysophosphatidylcholine, LPE = Lysophosphatidylethanolamine, PC = Phosphatidylcholine, PC O- = Ether-linked Phosphatidylcholine, PE O- = Ether-linked Phosphatidylethanolamine, PE = Phosphatidylethanolamine, PI = Phosphatidylinositol, SM = Sphingomyelin, TAG = Triacylglycerol.
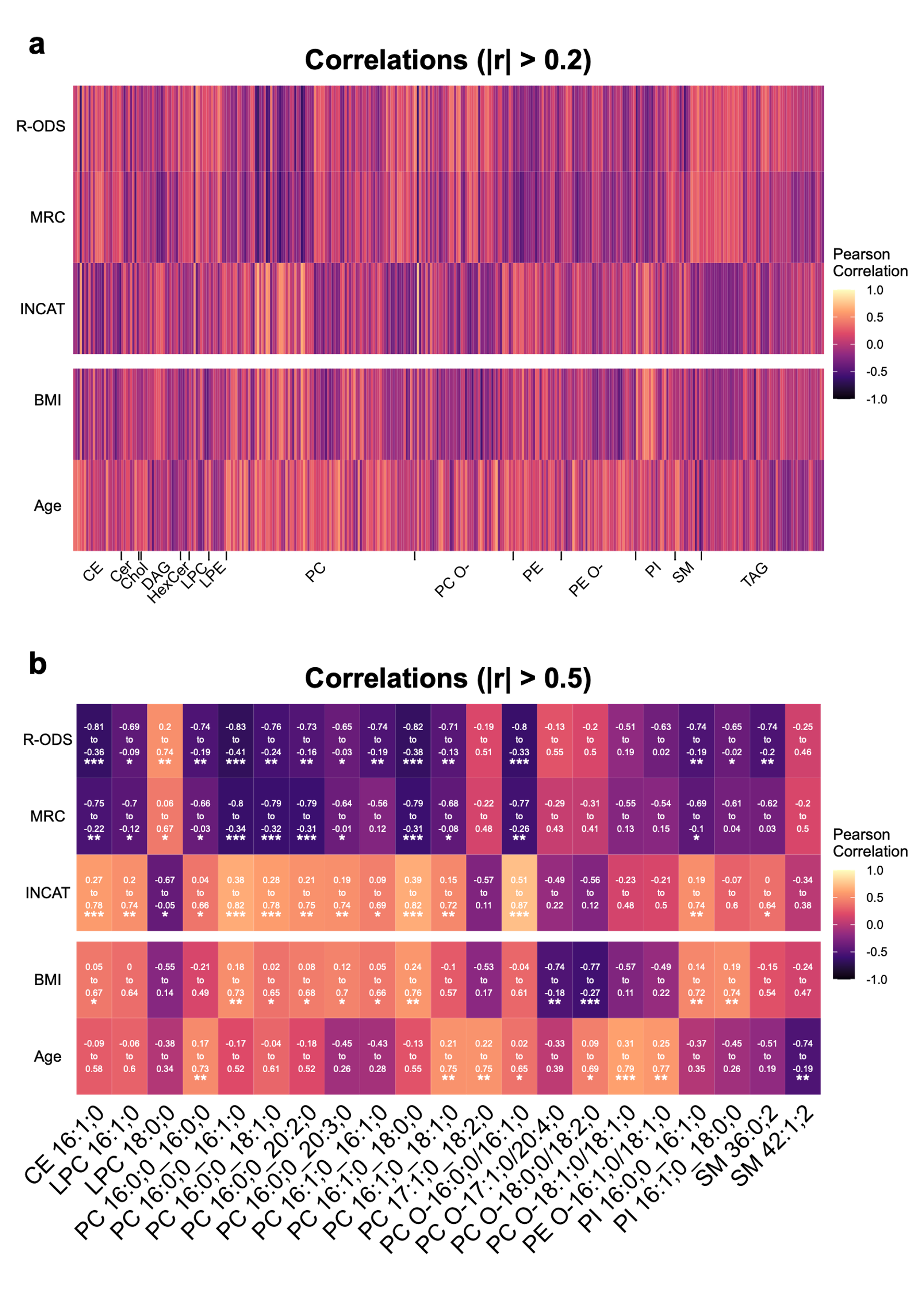


eFigure 7 Correlation Analysis.

Heatmap with color coding by Pearson correlation displaying all 343 lipids with an absolute correlation r > 0.2 (a) and 21 lipids with an absolute r > 0.5 (b). p < 0.05 = *; p < 0.01 = **; p < 0.001 = ***; Corresponding statistics in supplementary file 8.


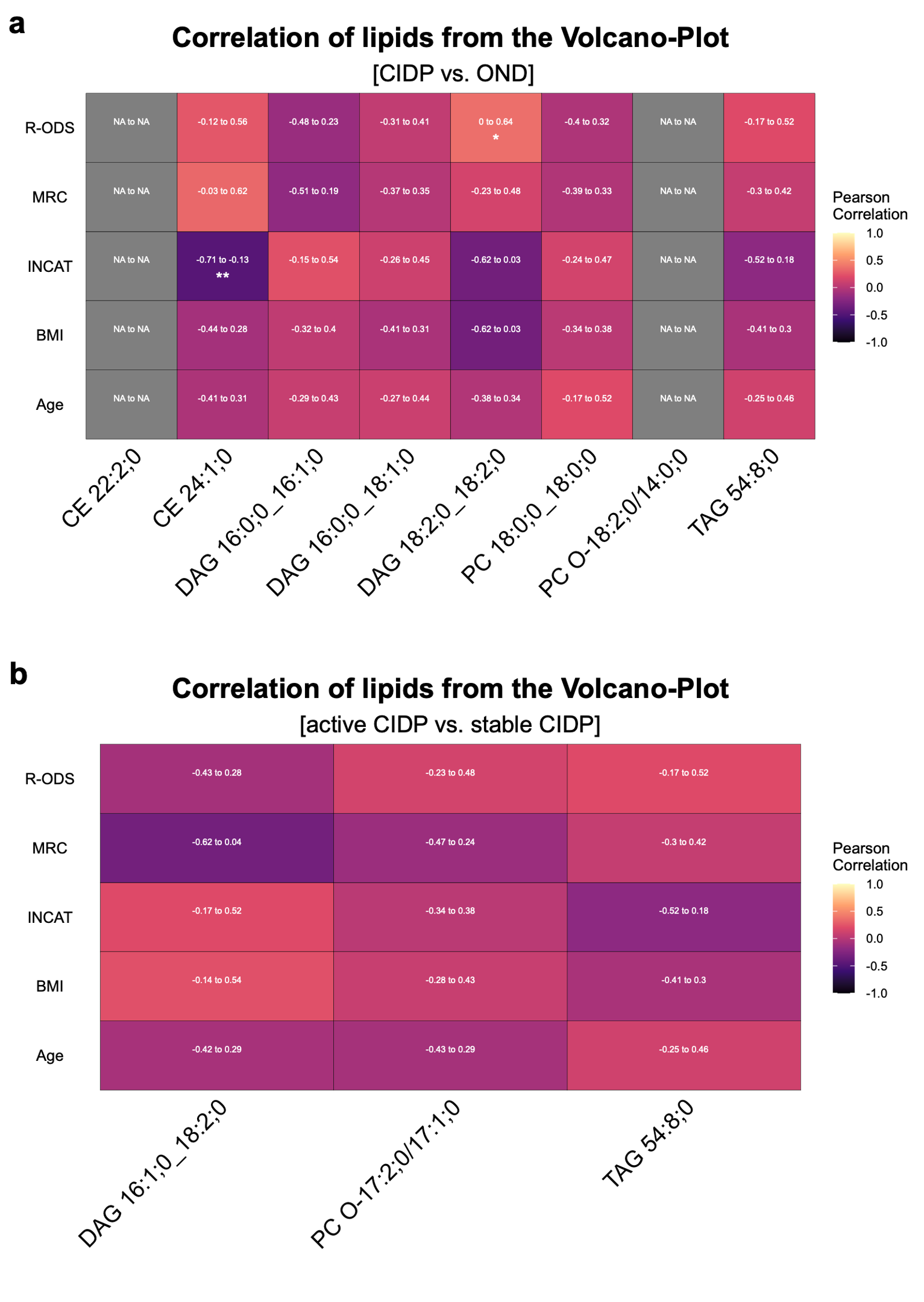


eFigure 8 Correlation of selected lipids.

Visualization of Pearson r correlation with the corresponding 95% confidence interval of r focusing on the statistically significant lipids from Figure 2 (**a**) and Figure 4(**b**). Statistically significant correlations are marked with * for p < 0.05 and ** for p < 0.01.

eFigure 9 - Raw mol % abundances of DAG (top) and TAG (bottom) species in CIDP versus OND (no imputation or transformation).

To highlight statistical findings from Figures 2 and 3, species names are colored as follows: Green: significant by Welch’s t-test (Figure 2); Red: significant by Welch’s t-test and |log₂ fold-change| > 1 (Figure 3). This raw-data view demonstrates that we capture both high- and low-abundance lipids.
